# Short-term effects of liraglutide on visceral fat adiposity, appetite, and food preference: a pilot study of obese Japanese patients with type 2 diabetes

**DOI:** 10.1186/1475-2840-10-109

**Published:** 2011-12-01

**Authors:** Kana Inoue, Norikazu Maeda, Susumu Kashine, Yuya Fujishima, Junji Kozawa, Aki Hiuge-Shimizu, Kohei Okita, Akihisa Imagawa, Tohru Funahashi, Iichiro Shimomura

**Affiliations:** 1 Department of Metabolic Medicine, Graduate School of Medicine, Osaka University, 2-2-B5, Yamada-oka, Suita, Osaka 565-0871, Japan; 2 Department of Metabolism and Atherosclerosis, Graduate School of Medicine, Osaka University, 2-2-B5, Yamada-oka, Suita, Osaka 565-0871, Japan

**Keywords:** liraglutide, glucagon-like peptide-1, obesity, eating behavior

## Abstract

**Background:**

To examine the effects of liraglutide, a glucagon-like peptide-1 (GLP-1) analogue, on visceral fat adiposity, appetite, food preference, and biomarkers of cardiovascular system in Japanese patients with type 2 diabetes.

**Methods:**

The study subjects were 20 inpatients with type 2 diabetes treated with liraglutide [age; 61.2 ± 14.0 years, duration of diabetes; 16.9 ± 6.6 years, glycated hemoglobin (HbA1c); 9.1 ± 1.2%, body mass index (BMI); 28.3 ± 5.2 kg/m^2^, mean ± SD]. After improvement in glycemic control by insulin or oral glucose-lowering agents, patients were switched to liraglutide. We assessed the estimated visceral fat area (eVFA) by abdominal bioelectrical impedance analysis, glycemic control by the 75-g oral glucose tolerance test (OGTT) and eating behavior by the Japan Society for the Study of Obesity questionnaire.

**Results:**

Treatment with liraglutide (dose range: 0.3 to 0.9 mg/day) for 20.0 ± 6.4 days significantly reduced waist circumference, waist/hip ratio, eVFA. It also significantly improved the scores of eating behavior, food preference and the urge for fat intake and tended to reduce scores for sense of hunger. Liraglutide increased serum C-peptide immunoreactivity and disposition index.

**Conclusions:**

Short-term treatment with liraglutide improved visceral fat adiposity, appetite, food preference and the urge for fat intake in obese Japanese patients with type 2 diabetes.

## Introduction

The prevalence of type 2 diabetes has rapidly increased worldwide [1] including Western and Asian countries [2]. Type 2 diabetes is a major risk for cardiovascular events. Obesity, especially visceral fat adiposity, also increases the risk of type 2 diabetes, hypertension, dyslipidemia, and atherosclerosis, suggesting that obese patients with type 2 diabetes are at high risk for cardiovascular diseases [3]. The World Health Organization (WHO) projects that 2.3 billion adults are overweight and > 700 million are obese [4]. The association between type 2 diabetes and overweight/obesity is indisputable. In this sense, it is necessary to develop effective and efficient therapeutic strategy for obese type 2 diabetes. However, the treatment for obese type 2 diabetes often encounters difficulties with regard to the control of weight and appetite. Moreover, treatment with insulin, sulfonylurea, and thiazolidinedione increase appetite and body weight, frequently resulting in poor glycemic control.

Dietary fat intake alters glucose and lipid metabolism [5] and correlates with cardiovascular risk in type 2 diabetes [6]. The intake of animal fat, especially saturated fat, is considered to be associated with type 2 diabetes and cardiovascular diseases [7]. Higher intake of polyunsaturated fat relative to saturated fat correlates negatively with the incidence of type 2 diabetes [8,9]. In addition to calorie restriction, the control of food preference is also another important factor in the treatment of obese type 2 diabetes.

Liraglutide is a glucagon-like peptide-1 (GLP-1) analogue with 97% structural homology to human GLP-1. Native GLP-1 has a short elimination half-life of 1-2 min, whereas liraglutide has a long half-life of about 13 hours and can be administered once a day [10]. GLP-1 is a naturally occurring incretin hormone with a potent blood-glucose lowering action only during hyperglycemia because it induces insulin secretion and reduces glucagon secretion in a glucose-dependent manner [11]. In addition, GLP-1 delays gastric emptying and induces satiety, leading to decreased energy intake and weight reduction. The underlying mechanisms of weight loss are most probably a combination of the effects of GLP-1 on the gastrointestinal tract and the brain [12]. Recent experiments show that the anorectic effect of peripheral GLP-1 administration is mediated both by the activation of GLP-1 receptor (GLP-1R) expressed on vagal afferents and by the GLP-1R activation in central nervous system [13]. GLP-1 also has various extrapancreatic actions such as the cardiovascular system [4] and is considered a promising new agent for the treatment of type 2 diabetes and cardiovascular diseases linked to obesity-type 2 diabetes.

In the present study, we investigated the effects of liraglutide on visceral fat adiposity, eating behavior, and cardiovascular biomarkers in controlled hospitalized patients with type 2 diabetes.

## Materials and methods

### Subjects

All enrolled subjects were hospitalized in the Division of Endocrinology & Metabolism, Osaka University Hospital. They represented inpatients with type 2 diabetes treated for the first time with liraglutide between August 2010 and April 2011. Upon admission, each patient was treated with insulin or oral glucose-lowering agents under diet therapy to improve plasma glucose levels. After achieving the target levels of glycemic control [fasting plasma glucose (FPG) < 150 mg/dL and postprandial 2-h plasma glucose < 200 mg/dL], treatment with insulin or oral glucose-lowering agents was replaced with liraglutide at 0.3 mg/day, which was increased by 0.3 mg/day every one week to a final dose of 0.9 mg/day, representing the maximum dose in Japan. Introduction of liraglutide was decided by each attending physician after confirmation of preserved β-cell function by the examination of fasting serum C-peptide, daily urine C-peptide, and/or, glucagon challenge test. Among the 20 study subjects, liraglutide treatment failed to lower FPG to the target levels even at 0.9 mg/day in 8 patients (sulfonylurea (SU), n = 4; biguanide (BG), n = 1; SU+BG, n = 3), necessitating the addition of oral glucose-lowering agents according to the assessment of the attending physician. Written consent was obtained from each subject after explaining the purpose and complications of the study. The study protocol was approved by the human ethics committee of Osaka University and was registered with the University hospital Medical Information Network (Number: UMIN 000004192).

### Clinical examination

Various metabolic parameters were measured on admission, liraglutide induction, and discharge. Waist circumference at umbilical level was measured in standing position with a non-stretchable tape in the late phase of expiration. Estimated visceral fat area (eVFA) was measured by abdominal bioelectrical impedance analysis (BIA), as described previously [14].

Several diabetic parameters were examined before and after liraglutide use. Each subject received a 75-g oral glucose tolerance test (OGTT) after 10- to 12-h overnight fast. Insulin secretion was evaluated by fasting serum C-peptide (F-sCPR), C-peptide index (CPI), insulinogenic index, and disposition index. Insulin resistance was assessed by homeostasis model assessment of insulin resistance (HOMA-IR) and the Matsuda index. These parameters were calculated by the following formula: CPI = F-sCPR (ng/mL) × 100/fasting glucose (mg/dL), insulinogenic index = [(insulin at 30 min) - (insulin at 0 min)]/[(glucose at 30 min) - (glucose at 0 min)], HOMA-IR = fasting glucose (mg/dL) × fasting insulin (μU/ml)/405, composite (Matsuda) index and disposition index were calculated using the MINMOD Millenium software [15,16]. High-sensitivity C-reactive protein (hsCRP) (N-Latex CRP II, Dade Behring Inc, Marburg, Germany) and soluble intercellular adhesion molecule-1 (sICAM-1) (Pierce Biotechnology, Rockford, IL) were also measured before and after liraglutide treatment.

To evaluate dietary intake, staple food and non-staple food were quantified at each meal by the nurses. Every intake of staple food and non-staple food was rated on an eleven-point scale ranging from 0 (no intake) to 10 (full intake), respectively. For example, if all staple food was consumed every meal, the daily score for staple food intake was 30 point/day. Scores were recalculated based on previous prescribed total daily calorie when the diet was changed. Food intake was quantified by the daily average intake from admission to liraglutide induction, and from liraglutide induction to discharge, respectively.

### Questionnaire for eating behavior

Eating behavior was assessed by using the questionnaire of The Guideline For Obesity issued by the Japan Society for the Study of Obesity (advocated by Prof. Hironobu Yoshimatsu), at admission, pre- and post-liraglutide treatment. This questionnaire consists of 55-item questions of seven major scales as follows: 1. Recognition for weight and constitution (e.g., 'Do you think it is easier for you to gain weight than others?', 'Do you think you gain weight because of less exercise?'), 2. External eating behavior (e.g., 'If food smells and looks good, do you eat more than usual?', 'If you walk past the supermarket, do you have the desire to buy something delicious?', 'If you see others eating, do you also have the desire to eat?'), 3. Emotional eating behavior (e.g., 'Do you have the desire to eat when you are irritated?', 'Do you have a desire to eat when you have nothing to do?'), 4. Sense of hunger (e.g. 'Do you get irritated when you feel hungry?', 'Do you often regret because you have eaten a lot of food?'), 5. Eating style (e.g., 'Do you eat fast?', 'Are you known to eat a lot of food?'), 6. Food preference (e.g., 'Do you often eat snack bread?', 'Do you like meat?', 'Do you like noodles?'), 7. Regularity of eating habits (e.g., 'Is your dinner time too late at night?', 'Do you gain body weight during holidays?'). All items were rated on a four-point scale ranging from 1 (seldom) to 4 (very often).

### Statistical analysis

Results are expressed as mean ± standard deviation (SD). All analyses were performed using JMP software (JMP 8.0; SAS Institute Inc., Cary, NC). For comparison of results obtained at admission, before and after liraglutide treatment, the Student's t-test was used.

## Results

### Characteristics of participants

Table 1 shows baseline characteristics of 20 subjects at admission. The mean age was 61.2 years, body mass index (BMI) 28.3 kg/m^2^, and the duration of diabetes was 16.9 years. The mean glycated hemoglobin (HbA1c) was 9.1%. At baseline, 65% of patients were treated with insulin (average dosage: 37.3 units/day, maximum dosage: 70 units/day). These parameters indicate that the enrolled patients had relatively long duration of diabetes, mild obesity, high frequency of insulin use, and high dosage of insulin.

**Table 1:** Baseline characteristics.


Sex (Male/Female)	8/12
Age (years)	61.2 ± 14.0
Duration of diabetes (years)	16.9 ± 6.6
HbA1c (%)	9.1 ± 1.2
Body mass index (kg/m^2^)	28.3 ± 5.2
Waist circumference (cm)	100.0 ± 11.8
Waist-to-hip ratio	1.01 ± 0.07
Estimated visceral fat area (cm^2^)	182.6 ± 68.3
LDL-C (mg/dL)	112.7 ± 29.1
HDL-C (mg/dL)	45.7 ± 8.8
Triglycerides (mg/dL)	141.6 ± 68.2
Uric acid (mg/dL)	6.0 ± 1.5
History of smoking (%)	30
Hypertension (%)	70
Dyslipidemia (%)	95
History of cardiovascular disease (%)	30
Medication for Diabetes	
Biguanide (%)	50
Sulfonylurea (%)	45
Alpha-glucosidase inhibitor (%)	30
Thiazolidinedione (%)	25
DPP-IV inhibitor (%)	10
Glinide (%)	10
Insulin (%)	65

Data are mean ± SD or number of subjects. LDL-C; low-density lipoprotein-cholesterol, HDL-C; high-density lipoprotein-cholesterol, DPP-IV; dipeptidyl peptidase-IV.

After admission, patients were started on insulin or oral glucose-lowering agents under diet therapy until glycemic control reached target levels, considered as relief from gluco-toxicity, and were then introduced to liraglutide treatment at 0.3 mg/day. The dose was gradually increased according to blood glucose levels as described in the Methods section. The number of patients treated with liraglutide at 0.3, 0.6, and 0.9 mg/day at discharge was 1, 9, and 10 patients, respectively. The time period from admission to induction of liraglutide was 11.1 ± 4.7 days and from induction to discharge was 20.0 ± 6.4 days.

### Changes in physical parameters and visceral fat adiposity

**Figure 1:**
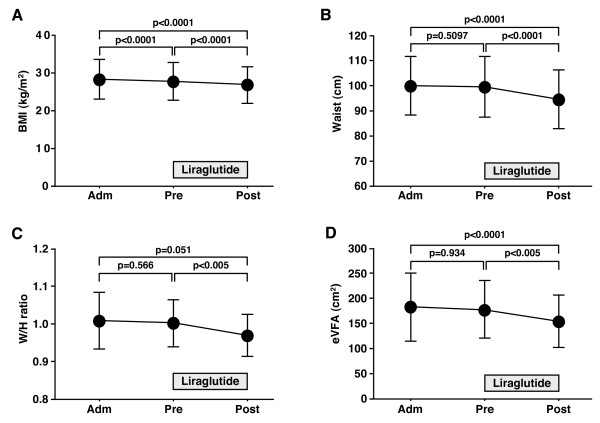
**Liraglutide-induced changes in visceral fat adiposity**. Data are mean ± SD of 20 patients. BMI, body mass index; W/H, waist/hip; eVFA, estimated visceral fat area; Adm, admission; Pre, pre-liraglutide treatment; Post, post-liraglutide treatment.

BMI was significantly reduced by 0.54 ± 0.11 kg/m^2 ^from admission to liraglutide induction with further significant decrease by 0.93 ± 0.12 kg/m^2 ^from liraglutide induction to discharge (Figure 1A). The waist circumference decreased by -0.30 ± 0.44 cm from admission to liraglutide induction, and liraglutide treatment significantly decreased waist circumferences by -4.28 ± 0.55 cm from liraglutide induction to discharge (Figure 1B). The waist/hip (W/H) ratio at admission, liraglutide induction, and discharge was 1.01 ± 0.07, 1.00 ± 0.06, and 0.97 ± 0.06, respectively, and was significantly reduced by liraglutide (Figure 1C). The eVFA at admission, liraglutide induction, and discharge were 182.6 ± 68.3 cm^2^, 178.1 ± 56.8 cm^2^, and 153.4 ± 51.9 cm^2^, respectively, (Figure 1D). Similar to waist circumference and W/H ratio, liraglutide significantly reduced eVFA compared with no significant change before liraglutide induction.

### Assessment of food intake, appetite, and food preferences

**Figure 2:**
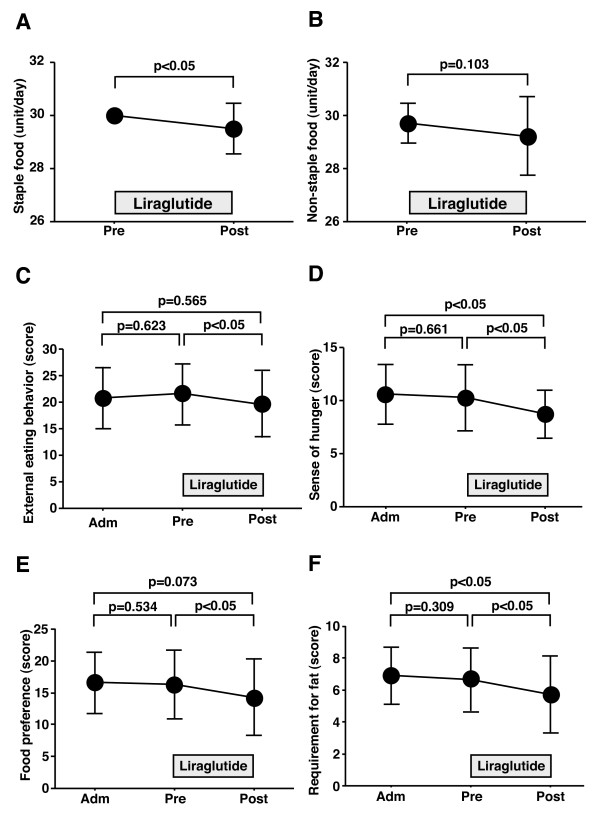
**Liraglutide-induced changes in food intake and appetite**. Every intake of staple food and non-staple food was rated on an eleven-point scale ranging from 0 (no intake) to 10 (full intake), respectively. Eating behavior was assessed by using a questionnaire as described in method section. Data are mean ± SD of 20 patients. For abbreviation; see Figure 1.

Next, the effects of liraglutide on food intake, appetite, and food preference were evaluated. Liraglutide significantly decreased daily staple food intake (Figure 2A), but did not change significantly the daily intake for non-staple food (Figure 2B). Patients received a questionnaire on eating behavior at admission, pre- and post-liraglutide as described in the Methods section. Figure 2C to 2E show changes in the scores for representative question headings; external eating behavior, sense of hunger, and food preference. These score did not change under conventional glucose-lowering treatment, i.e., before liraglutide induction. However, treatment with liraglutide significantly decreased the scores for external eating behavior (Figure 2C) and tended to decrease the sense of hunger (Figure 2D). The number of patients who reported feeling full stomach was higher after liraglutide induction. Interestingly, liraglutide administration also decreased the score of food preference, related to the obesity phenotype (Figure 2E), and especially reduced the need for fat intake such as fatty foods, meat diet, and snack breads (Figure 2F).

### Effect of liraglutide treatment on glucose tolerance

OGTT was conducted just before liraglutide induction, i.e., after relief of gluco-toxicity. Table 2 lists various indices related to insulin sensitivity and insulin secretion measured before and after induction of liraglutide treatment. There were no significant differences in HOMA-IR and the Matsuda index between pre- and post-liraglutide, while fasting serum C-peptide immunoreactivity (F-sCPR) and C-peptide index (CPI) were significantly elevated by liraglutide treatment. Liraglutide also increased the insulinogenic index and disposition index. These data indicate that liraglutide ameliorates insulin secretion as expected.

**Table 2:** Liraglutide-induced changes in parameters relating to glucose metabolism.

	Before treatment with liraglutide	After treatment with liraglutide	P value
sCPR(ng/mL)	1.87 ± 1.00	3.25 ± 1.19	< 0.0001
C-peptide Index	1.47 ± 0.75	2.76 ± 0.97	< 0.0001
HOMA-IR	3.52 ± 2.63	4.51 ± 3.27	0.468
Matsuda Index	3.09 ± 2.24	3.16 ± 1.99	0.820
Insulinogenic Index	0.18 ± 0.16	0.32 ± 0.30	0.058
Disposition Index	0.43 ± 0.27	0.76 ± 0.51	< 0.05

Data are mean ± SD. sCPR; serum C-peptide immunoreactivity, HOMA-IR; homeostasis model assessment of insulin resistance.

### Effect of liraglutide treatment on biomarkers of cardiovascular system

We also tested the effects of short-term liraglutide treatment on biomarkers of cardiovascular system. Serum hsCRP levels were significantly reduced by liraglutide (1.10 ± 1.03 mg/L at liraglutide induction versus 0.70 ± 0.47 mg/L at discharge, *P *< 0.05). Treatment with liraglutide tended to decrease plasma concentrations of sICAM-1 (198.23 ± 84.95 ng/mL at liraglutide induction versus 180.99 ± 59.27 ng/mL at discharge, *P *= 0.067).

## Discussion

The present study demonstrated that short-term liraglutide treatment significantly affected visceral fat adiposity, appetite, food preference, and cardiovascular biomarkers in Japanese patients with type 2 diabetes.

The LEAD-2 trial showed that liraglutide at 1.2 and 1.8 mg/day significantly decreased waist circumference and eVFA compared to the 4 mg/day glimepiride after 26 weeks from introduction, while there was no significant difference in the reductions of waist circumference and eVFA between liraglutide group and placebo group [17]. In the LEAD-3 substudy, a long-term liraglutide monotherapy significantly reduced body weight and fat mass from baseline while body weight and fat mass were increased in the glimepiride group [18]. In the present study, waist circumference, W/H ratio, and eVFA did not change while patients were treated with insulin and/or oral glucose-lowering agents (Figure 1). However, treatment with liraglutide significantly reduced these physical parameters. Furthermore, the daily reduction of waist circumferences was significantly larger during liraglutide treatment than before liraglutide introduction, suggesting that liraglutide effectively decreased visceral fat adiposity. Previous studies reported the lack of GLP-1 receptor expression on adipocytes [19], but recent work has demonstrated GLP-1 receptor expression on adipocytes [20]. Although GLP-1 action and GLP-1 receptor signaling in adipocytes have not been fully elucidated, the present study demonstrated that liraglutide reduced visceral fat adiposity in a direct or indirect fashion. The results also point to a possible anti-atherosclerotic effect for liraglutide partly through reduction of eVFA. Treatment with exenatide, another GLP-1 receptor agonist, exhibited a significant weight reduction when used for 3 years [21]. Previous study showed that body weight did not change following the use of liraglutide by Japanese type 2 diabetes patients, but the mean BMI of those patients was only 23.9 kg/m^2 ^[22]. As shown in Table 1 the mean BMI of our patients was 28.3 kg/m^2^, suggesting that the effect of liraglutide on weight reduction is limited to obese type 2 diabetics.

Several studies have demonstrated that GLP-1 promotes satiety and suppresses energy intake both in animals [23,24] and human subjects [25-28], however, the effect of liraglutide on eating behavior has not been examined in human type 2 diabetics. The present study demonstrated that liraglutide reduced food intake and changed external eating behavior and food preference. Liraglutide significantly reduced the intake for staple food, e.g., rice and/or bread. Interestingly, liraglutide significantly decreased the scores for external eating behavior and food preference (Figure 2C and 2E). Questions on external eating behavior evaluate appetite increase through sight and smell senses. Questions about food preference measure foods directly associated with obesity. Such improvement in eating behavior induced by liraglutide has not been reported for other glucose-lowering agents. Furthermore, liraglutide significantly reduced the urge for fat intake (Figure 2F). Although the findings of epidemiological studies on the association of total dietary fat with type 2 diabetes have been inconsistent [29-32], dietary fat intake impairs glucose metabolism [5] and is strongly related to cardiovascular risk in type 2 diabetes [6]. A high intake of saturated fat is considered to associate with type 2 diabetes and cardiovascular diseases [7]. In this sense, liraglutide may be beneficial in reducing fat preference, related to obese type 2 diabetes, although the long-term effects of liraglutide on eating behavior remain to be elucidated. It would be better to refine the questions on food preference to identify the types of fat.

GLP-1 receptor agonist exerts glucose-lowering effect mainly by stimulating glucose-mediated secretion of insulin from β-cells. Insulin secretion is often deteriorated in various degrees in Japanese and the other Asian patients with type 2 diabetes compared to Caucasian [2], and thus the effect of liragulutide on insulin secretion is worth evaluating in Japanese type 2 diabetes subjects. As shown in Table 2 liraglutide treatment increased sCPR and C-peptide Index, indicating that insulin secretion was enhanced by liraglutide. Such changes may appear the increases of HOMA-IR and Matsuda Index, suggesting that these indices may not exactly reflect insulin sensitivity under liraglutide treatment. There is a possibility that long-term liraglutide treatment may ameliorate insulin resistance by weight reduction through decrease of appetite. Further clinical investigations may be needed in future.

The extrapancreatic actions of GLP-1 have been demonstrated, especially the beneficial its effect on the cardiovascular system [4]. GLP-1 has a protective action on heart function and/or cardiomyocytes, although the GLP-1 action on the vascular system has not been fully evaluated especially in human. Treatment with GLP-1 improved endothelial dysfunction in type 2 diabetics with ischemic heart diseases [33]. Interestingly, GLP-1 treatment improved postprandial hyperlipidemia, suggesting the possibility that GLP-1 administration may reduce cardiovascular disease risk in type 2 diabetes [34]. A recent experiment showed that GLP-1 treatment protected against the development of atherosclerosis both *in vivo *and *in vitro *[35,36]. In the present study, liraglutide decreased serum hsCRP and sICAM-1 levels, while no such changes were observed following treatment with other glucose-lowering agents with insulin (n = 6), biguanide (BG) (n = 4), α-glucosidase inhibitor (αGI) (n = 3), dipeptidyl peptidase-4 inhibitors (DPP-4i) (n = 3), and sulfonylurea (SU) (n = 2) (data not shown). These results suggest that the decrease in hsCRP and sICAM-1 levels may be due to the pleiotropic effects of liraglutide, beneficial for protection against the development of atherosclerosis.

The present study has several limitations. The study is not a randomized clinical trial (RCT) and is not a crossover study. A crossover clinical trial will confirm present results. In addition, present study was performed in a small population. The effects of liraglutide on visceral fat adiposity, appetite, and cardiovascular biomarkers were examined in a hospitalized term. Collectively, a long-term RCT in obese Japanese type 2 diabetics should be conducted to confirm the efficacy of liraglutide on visceral fat adiposity, appetite, food behavior, and cardiovascular events.

In summary, short-term treatment with liraglutide effectively reduced visceral fat adiposity, appetite, and cardiovascular biomarkers in obese Japanese patients with type 2 diabetes. Longer term randomized clinical trials are warranted to more thoroughly elucidate the effect of liraglutide on these parameters.

## Competing interests

The authors declare that they have no competing interests.

## Authors' contributions

KI acquired and analyzed data, and wrote the manuscript. NM conceived study, analyzed data, and wrote the manuscript. SK, YF, JK, AHS, KO, and AI acquired and researched data. TF and IS reviewed manuscript. All authors read and approved the final manuscript.

## Acknowledgements

We thank Miyuki Nakamura, Department of Metabolic Medicine, Graduate School of Medicine, Osaka University, for the excellent technical assistance. This work was supported in part by Grants-in-Aid for Scientific Research (C) no. 22590979 (to N. M.) and Scientific Research on Innovative Areas no. 22126008 (to T. F.). This article is dedicated to the memory of Dr. Hironobu Yoshimatsu, who was a professor of Oita University and proposed the questionnaire of eating behavior.
